# Relative handgrip strength, nutritional status and abdominal obesity in Chilean adolescents

**DOI:** 10.1371/journal.pone.0234316

**Published:** 2020-06-10

**Authors:** Ana Palacio-Agüero, Ximena Díaz-Torrente, Daiana Quintiliano Scarpelli Dourado

**Affiliations:** Escuela de Nutrición y Dietética, Facultad de Medicina-Clínica Alemana, Universidad del Desarrollo, Santiago, Chile; University of Houston, UNITED STATES

## Abstract

Handgrip strength (HGS) is a well-established indicator of muscle strength and can help to identify risk of sarcopenic obesity in children. This study explores the relationship between adiposity and muscular strength in healthy Chilean adolescents. Adolescents (n = 491) aged 10–17 were selected from five schools in Santiago, Chile. HGS was determined by dynamometry. Anthropometry (weight, height, waist and mid arm circumference), physical activity and socioeconomic status were also measured. Relative HGS (RHGS) was calculated by dividing maximum HGS of the dominant hand by body-mass index (BMI) and low RHGS was categorized as <25^th^ percentile by sex. Logistic regression was used to determine the relationship between two markers of adiposity (abdominal obesity category by waist circumference and nutritional status measured by BMI category) and low RHGS, adjusting for possible confounding variables. Participants were on average 13.6y (2.4), 32.8% were overweight or obese and 37.5% were at risk of or had abdominal obesity. RHGS was 1.25 kg/kg/m^2^ overall, with a significant difference by sex (1.51 for boys versus 1.14 for girls). In adjusted analyses, boys and girls with risk of abdominal obesity, had 3.3 (1.6–6.6) and 4.1 (1.8–9.3) increased odds of low RHGS, respectively, compared to boys and girls with normal waist circumference. Those with abdominal obesity compared to normal WC, had 8.5 (3.4–21.4) and 6.5 (2.0–21.3) increased odds of low RHGS for boys and girls, respectively. We observed similar associations for BMI category. In our sample of healthy adolescents, higher adiposity related to greater odds of low muscle strength measured by dynamometry. Considering the demographic shift from a young to an aging population in many countries, along with the increasing prevalence of obesity beginning in childhood, understanding how adiposity relates to low muscle strength is of growing importance.

## Introduction

Muscle strength has been studied in different populations in relation to individual variables (sex, age, handedness) and with other factors that, similar to strength, are also sensitive to growth and development, such as weight, height, and body mass index (BMI) [1]. Starting in adolescence, there are notable differences in muscle strength by sex, reflecting an important sexual dimorphism that begins in this developmental period [2]. Overall variability is attributed to body composition, developmental stage, and environmental or lifestyle factors, such as physical activity [3,4].

Muscular strength is often measured via handgrip strength (HGS). The AVENA (Food and Assessment of the Nutritional Status of Spanish Adolescents) [5], HELENA (Healthy Lifestyle in Europe by Nutrition in Adolescents) [6] and ALPHA (Assessing Levels of Physical Activity and fitness) [7] studies have shown that HGS and the horizontal jump are the most used tests in epidemiological studies to assess muscle condition in children and adolescents, due to the high degree of reliability and validity. Using HGS to measure muscle condition has many advantages. For example, compared to tools such as dual energy x-ray absorptiometry and bioelectrical impedance, HGS can be measured quickly and easily in field-based testing [8].

Several studies have focused on the relationship between HGS and stature or body weight [9–12], and new evidence has focused on the importance of expressing HGS as a relative value (per m^2^ of body surface). Specifically, one study has suggested using relative HGS (RHGS)—HGS divided by BMI, as it has been strongly correlated with cardiovascular biomarkers [8]. RHGS may be especially important when evaluating children and adolescents, in order to account for differences in development and body size.

In addition of being a proxy for muscular strength, HGS and RHGS has been proposed as a tool sensitive for use in patients with neuromuscular disorders [13], and for identifying cardiometabolic health [14], including the metabolic syndrome [15], and sarcopenia in the elderly [16–18]. Sarcopenia, the loss of muscle mass and function, and dynapenia, low muscle mass and power, have been most frequently associated with old age; however, recent research has shown that even children are at risk [19], due to the current epidemic of sedentary lifestyles [20,21]. Nutritional status is an important consideration in the development of sarcopenia, as obesity seems to contribute to the development of sarcopenia resulting in what is called “sarcopenic obesity” [22]. Both have similar inflammatory pathways and may potentiate each other. Recent studies have demonstrated that sarcopenic obesity is associated with an increased risk of physical disability, cardiovascular morbidity and mortality when compared to either sarcopenia or obesity alone [18]. Adiposity also impairs muscle functionality through intramuscular fat deposition resulting in decreased muscle mass, strength and disability [23].

Life expectancy has significantly increased across Latin America in the last 50y [24]. In Chile, life expectancy has increased by 17y; from 63.5 in 1970 to 80.2 in 2020 [25]. Thus, sarcopenia will continue to be a public health priority for Chile and other countries with similarly aging populations. Considering that Chile also has the highest prevalence of childhood obesity in South America (23% of schoolchildren and 13.2% of adolescents between 15 and 19y are obese) [26], understanding the link between obesity and risk of low muscle strength, as a proxy for future sarcopenia, is important. This study explores the relationship between adiposity and low muscle strength. Specifically, we quantify the association of two measures of adiposity (abdominal obesity and BMI-for-age category) with HGS and RHGS in a sample of healthy Chilean adolescents.

## Materials and methods

### Settings and participants

Using a cross-sectional study design, 491 adolescents between 10 and 17y from five schools (three private and two public) were selected via non-probabilistic sampling from four different municipalities in the Metropolitan Region of Santiago, Chile. Between May and October 2018, parents of adolescents were informed of the study objectives during parent/guardian meetings at participating schools and invited to review the study protocol. Exclusion criteria included: hand surgery or injury in the last 6 months, any limitation in the use of both hands that could constrain the proper use of the dynamometer, being pregnant, and not being born in Chile. Parent/guardian informed consent and adolescent informed assent were obtained. All study procedures took place during physical education classes at adolescents’ schools. The study protocol followed the Helsinki Declaration and was approved by the Ethics Committee of the Universidad del Desarrollo–Facultad de Medicina Clínica Alemana.

### Measurements

#### Socioeconomic level

Parents reported total household income and the number of persons regularly living in the household. Socioeconomic level was determined by dividing income by number living in the household according to Chilean standards [27].

#### Physical activity

Type of activity, frequency and duration was self-reported by adolescents. Physical activity type was later classified by the research team in the following categories according to intensity and predominant oxygen use pathway: aerobic (cycling, dancing, running and endurance training), anaerobic (resistance training, weight lifting, yoga and archery) or mixed exercises (ballet, soccer, basketball, handball, volleyball, rhythmic gymnastics, swimming, etc.) [28]. Those who reported not engaging in regular physical activity were classified as sedentary.

#### Handgrip strength

Handgrip dynamometry was performed by trained evaluators during physical education classes using a Jamar^®^ Digital Grip Strength Dynamometer, which measures isometric grip strength in kilograms of force (kgf), with an accuracy of 0.1 kgf. The measurement protocol of The American Society of Hand Therapists was used [29]. The evaluator performed a demonstration of the correct grip strength measurement before the test. Participants were seated with forearms and wrists resting on their thighs. Participants took the dynameter in the second position with elbows flexed to 90 degrees and were instructed to tighten the dynamometer with maximum force after receiving a verbal order. Starting hand was alternated between subjects irrespective of the handedness. Every subject was encouraged to achieve maximum strength. The process was repeated 3 times for each hand with at least 60s rest between attempts for the same hand. Participants self-reported their dominant hand (right hand, left hand, or ambidextrous). The highest value obtained from the dominant hand was considered the maximum HGS.

#### Anthropometric measures

Anthropometric measures (weight, height, waist circumference (WC), and mid arm circumference) were conducted by trained evaluators according to the National Health and Nutrition Examination Survey protocol [30]. Participants were weighed using a SECA 803^®^ scale with 100g precision in light clothing and bare feet. Height was measured in the Frankfurt position using a SECA 213^®^ stadiometer with 1 mm precision. WC and mid arm circumference were measured in centimeters with a Seca 201^®^ non-elastic flexible tape with 1 mm precision. For WC measurement the reference point used was above the uppermost lateral border of the right ilium.

BMI was calculated in kg/m^2^ using measured weight and height. Participants were classified as “underweight,” “normal weight,” “overweight,” or “obesity” according to z-score criteria of the World Health Organization [31]. Abdominal obesity was classified as “normal”, “risk of abdominal obesity” “abdominal obesity” according to the guidelines for WC from the Chilean Ministry of Health [32]. Relative hand grip strength (RHGS) was calculated by dividing maximum HGS from the dominant hand by BMI. We defined low RHGS as <25^th^ percentile by sex.

### Statistical analysis

Descriptive data from qualitative variables were expressed in relative and absolute frequencies. Quantitative data were described as medians, interquartile ranges and percentiles (5, 10, 25, 50, 75, 90, 95), according to sex and dominant hand. The Shapiro-Wilk test was used to determine the normality of the distribution. For bivariate analysis assessing the sex differences in covariates, the chi-square, Kruskal-Wallis and Mann-Whitney U tests were used depending on variable type. For bivariate associations between maximum HGS and adiposity, Kruskal-Wallis and Mann-Whitney U tests were used. To compare differences in maximum HGS within categories of nutritional status and abdominal obesity, the post-hoc Dunn’s multiple-comparison test was used. Multivariate logistic regression was used to assess the association between two measures of adiposity (nutritional status and abdominal obesity) and low RHGS, stratified by sex. The variables included in the multivariate model were those with p<0.20 in the univariate model. The fitted modeling procedure was stepwise forward. Multivariable models were adjusted for the following covariates: socioeconomic level, age and physical activity, independent of its significant level. In final multivariable models, covariates with p>0.05 or multicollinearity (variance inflation factor >4) were removed for parsimony. Goodness of fit was measured with the Hosmer-Lemeshow test. Alpha was set at p <0.05. Statistical analysis was carried out using STATA 14.1 for Windows.

## Results

Participants were on average 13.6y (2.4). Table 1 shows the general description of the sample overall and by sex. More than half (71.4%), belonged to families with a low-to-medium socioeconomic level (<US$ $1,186 per capita). In relation to weight and height, boys were larger than girls: 55.3 kg and 1.66 m vs. 51.0 kg and 1.56 m, respectively (p<0.001 for sex difference). The majority of the sample (60.8%) had a normal nutritional status, with no statistically significant difference by sex (59.1% of boys and 62.6% of girls). There were no significant differences in mid arm circumference nor in abdominal obesity category, however, boys had a larger WC than girls (75 vs 71 cm, p< 0.001). Nearly a third of participants (30.8%) were sedentary. No significant differences were observed in the type of physical activity performed by sex. Although 52 different types of activities were identified, mixed activities were the most commonly reported. Specifically, the three most frequently reported mixed activities were soccer (17.8%), basketball (10.5%) and resistance training (7.1%).

**Table 1:** Description of the sample by sex.

Variables	Overall (n = 491)	Boys (n = 254)	Girls (n = 237)	p-value^a^
**Income per capita, % (n)**				0.092
US$ ≥ 2,078	11.2 (50)	14.4 (34)	7.6 (16)	
US$ 2,077–1,185	17.4 (78)	17.3 (41)	17.5 (37)	
US$ 1,186–381.0	42.9 (192)	43.1 (102)	42.7 (90)	
US$ ≤ 380	28.5 (128)	25.3 (60)	32.2 (68)	
**Weight (kg)**^b^	53.0 (43.5–61.5)	55.3 (45–65)	51.0 (42.0–58.0)	<0.001
**Height (m)**^b^	1.60 (1.51–1.68)	1.66 (1.53–1.73)	1.56 (1.48–1.61)	<0.001
**BMI (kg/m**^**2**^**)**^b^	20.4 (18.4–22.7)	20.4 (18.3–22.7)	20.3 (18.4–22.7)	0.776
**Nutritional status, % (n)**				0.325
Underweight	6.4 (31)	8.3 (21)	4.3 (10)	
Normal weight	60.8 (296)	59.1 (149)	62.6 (147)	
Overweight	23.8 (116)	23.8 (60)	23.8 (56)	
Obesity	9.0 (44)	8.7 (22)	9.4 (22)	
**Mid arm circumference (cm)**^b^	25.6 (23.0–28.0)	26.0 (23.0–28.2)	25.4 (23.0–27.4)	0.063
**WC (cm)**^b^	74.0 (68.0–80.0)	75.0 (70.5–82.0)	71.0 (66.2–77.5)	<0.001
**Abdominal obesity, % (n)**				0.435
Normal WC	62.5 (305)	62.9 (159)	62.1 (146)	
At risk	27.5 (134)	25.7 (65)	29.4 (69)	
Abdominal obesity	10.0 (49)	11.5 (29)	8.5 (20)	
**Right handedness, % (n)**	89.4 (439)	87.0 (221)	92.0 (218)	0.073
**HGS**				
Dominant hand (kg)^b^^,^^c^	26.9 (20.2–33.7)	31.5 (21.6–39.0)	24.0 (19.3–28.3)	<0.001
Right hand (kg)^b^^,^^d^	25.3 (18.6–31.7)	29.0 (20.0–37.2)	22.6 (17.8–26.7)	<0.001
Left hand (kg)^b^^,^^d^	23.3 (17.7–29.5)	27.2 (19.2–35.4)	21.2 (16.9–24.9)	<0.001
RHGS (kg/kg/m^2^)^b^	1.25 (0.99–1.62)	1.51 (1.09–1.84)	1.14 (0.96–1.33)	<0.001
**Physical activity, % (n)**				0.436
Sedentary	30.8 (151)	27.6 (70)	34.2 (81)	
Aerobic	12.6 (62)	12.6 (32)	12.7 (30)	
Anaerobic	3.1 (15)	3.2 (8)	2.9 (7)	
Mixed	53.6 (263)	56.7 (144)	50.2 (119)	

Abbreviations: BMI: body mass index; WC: waist circumference; HGS: handgrip strength; RHGS: relative handgrip strength.

^a^ Chi-square or Mann-Whitney U test depending on variable type for sex difference.

^b^ Variables are described as median and interquartile range.

^c^ Maximum.

^d^ Mean of 3 measurements.

The majority of participants reported being right-handed: 87% of boys and 92% of girls. Boys had greater handgrip strength in both hands compared to girls (29.0 and 27.2 kg for boys vs 22.6 and 21.2 kg for girls for the right and left hand, respectively).

Descriptive statistics of handgrip strength values in both hands by sex and age are provided in Table 2. In all cases, higher measures of strength were registered for the right hand versus the left. The median value (50^th^ percentile) ranged from 17.9 to 36.8 kg for boys and 17.5 to 27.4 kg for girls. In both sexes, HGS increased with age. Boys and girls who were 10-11y and 12-13y had similar median values of HGS; meanwhile HGS began to diverge by sex from 14y, with higher values for boys compared to girls.

**Table 2:** Percentile values of maximum strength (kg) of right and left hand by sex and age group in Chilean adolescents.

Age group	n	P5		P10		P25		P50		P75		P90		P95	
		RH	LH	RH	LH	RH	LH	RH	LH	RH	LH	RH	LH	RH	LH
**Boys**															
**10–11y**	54	12.7	11.7	13.2	12.7	15.1	14.8	17.9	16.8	20.4	18.7	23.7	21.6	30.6	27.7
**12–13y**	57	17.9	16.3	18.7	17.8	20.4	20.7	24.8	23.6	29.5	23.7	35.4	32.3	40.2	39.9
**14-15y**	46	28.7	24.5	29.2	26.1	32.9	30.1	35.8	34.8	40.3	38.6	46.8	43.0	49.2	44.4
**16-17y**	97	27.0	24.7	28.3	26.2	32.0	30.0	36.8	36.2	45.9	41.7	49.3	46.5	52.5	49.5
**Total**	254	15.0	14.7	17.2	16.2	21.7	20.8	31.5	28.9	38.5	36.9	46.6	43.0	49.2	46.2
**Girls**															
**10–11y**	79	11.8	10.9	13.7	12.3	14.7	14.2	17.5	16.1	20.0	19.6	26.1	23.7	27.0	25.6
**12–13y**	36	17.2	17.6	19.9	18.0	21.6	20.1	24.1	23.2	27.3	26.1	29.3	28.7	39.5	36.8
**14-15y**	25	20.2	18.9	20.7	19.1	24.6	23.5	27.8	25.4	31.3	27.7	35.3	32.1	35.9	33.8
**16-17y**	97	20.6	18.8	22.3	20.3	24.1	22.9	27.4	25.8	30.9	28.4	34.7	32.7	38.3	33.9
**Total**	237	13.9	13.4	15.3	14.8	19.3	18.2	24.1	22.9	28.3	26.2	32.3	30.1	35.9	32.9

Abbreviations: P: percentile; RH: right hand; LH: left hand.

Table 3 shows maximum HGS of the dominant hand, by adiposity (nutritional status and abdominal obesity) and age (group and overall), stratified by sex. Among boys, we observed an overall difference in HGS by category of adiposity (p = 0.017 for nutritional status and p = 0.043 for abdominal obesity). The difference in HGS by nutritional status was apparent among boys 14-17y (p = 0.008), but not statistically significant for boys 10-13y; HGS by abdominal obesity status was not significantly different within age categories. For nutritional status, post-hoc analyses revealed significant differences between the underweight group compared to all others. A similar pattern of associations was observed for the relationship between HGS and nutritional status for age categories for abdominal obesity, those with normal WC had significantly higher HGS compared to those at-risk.

**Table 3:** Maximum strength of dominant hand by age group, nutritional status and abdominal obesity, stratified by sex.

Age group	Nutritional status				Overall p-value	Abdominal obesity			Overall p-value
	Underweight	Normal weight	Overweight	Obesity		Normal	At risk	Obesity	
**Boys**	**n = 21**	**n = 149**	**n = 60**	**n = 22**		**n = 159**	**n = 65**	**n = 29**	
**10–13 (n = 111)**	17.9 (16.6–20.2)	21.6 (17.9–27.0)	20.0 (17.5–23.2)	24.3 (19.8–29.9)	0.069	20.3 (16.9–25.7)	20.5 (18.3–26.9)	21.2 (18.8–27.8)	0.510
**14–17 (n = 143)**	29.6^a^ (28.8–34.2)	38.0^b^ (33.2–43.9)	38.5^b^ (32.6–46.3)	37.8^b^ (29.8–45.4)	0.008	38.0 (32.5–43.8)	35.0 (32.1–42.2)	34.7 (30.0–48.5)	0.651
**Total (n = 254)**	27.4^a^ (18.0–30.0)	33.2^b^ (24.5–39.7)	26.8^a,c^ (19.7–39.8)	29.8^b,c^ (23.7–40.2)	0.017	32.8^a^ (24.0–40.1)	29.0^b^ (20.3–35.1)	28.6^a,b^ (20.0–34.7)	0.043
**Girls**	**n = 10**	**n = 147**	**n = 56**	**n = 22**		**n = 146**	**n = 69**	**n = 20**	
**10–13 (n = 115)**	15.7^a^ (12.6–17.3)	18.6^a^ (15.6–22.2)	20.6^b^ (18.4–25.5)	21.8^b^ (18.1–26.3)	0.024	20.2 (15.7–24.2)	19.3 (15.6–21.7)	19.3 (16.4–23.3)	0.626
**14–17 (n = 122)**	21.7^a^ (20.7–23.3)	27.4^b^ (24.2–30.9)	28.1^b^ (25.9–33.1)	28.3^b^ (26.8–31.9)	0.018	27.4 (24.1–30.9)	27.9 (25.3–31.6)	31.9 (26.7–32.5)	0.473
**Total (n = 237)**	20.0 (15.7–23.3)	24.0 (19.0–28.4)	25.9 (20.4–29.2)	24.8 (19.3–28.3)	0.090	25.8^a^ (21.3–28.9)	21.4^b^ (17.5–26.4)	20.6^b^ (16.7–29.2)	0.002

Variables are described as median and interquartile range.

Kruskal-Wallis test.

Different subscript letters represent significant differences (p<0.05), while same letters represent no statistical differences by Dunn’s multiple comparison test.

In the case of girls, significant differences were observed within age group for nutritional status and overall for abdominal obesity. Specifically, among young girls (10-13y), those who were underweight had the lowest maximum HGS compared to all other nutritional status classification: 15.7 versus 18.6, 20.6, and 21.8 for underweight, normal weight, overweight, and obese, respectively. This difference was statistically significant for comparisons between underweight, overweight and obesity. Similar relationships were found for girls at 14-17y. For abdominal obesity, girls with normal WC had higher maximum HGS compared to those at risk or with abdominal obesity (Dunn’s post-hoc test, p<0.05) (see Table 3).

**Fig 1:**
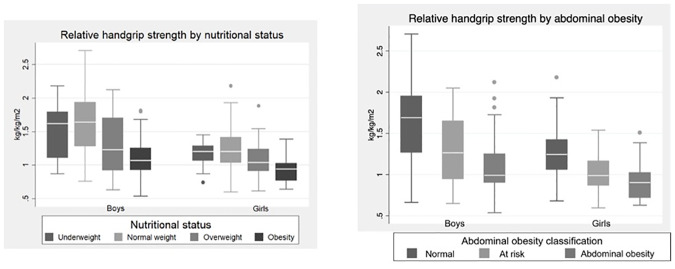
Relative handgrip strength (kg/kg/m^2^) by nutritional status and abdominal obesity category among Chilean adolescents.

RHGS was calculated and analyzed by nutritional status and abdominal obesity (Fig 1). For both sexes, lower values of RHGS were observed in the highest categories of adiposity (obesity and abdominal obesity), with statistically significant differences observed between normal nutritional status classification and obesity (p<0.001), and between normal WC and abdominal obesity (p<0.001).

Multivariable logistic regression analyses of the associations between low RHGS and category of adiposity, stratified by sex, are summarized in Table 4. For nutritional status, being underweight compared to normal weight between both boys and girls was not associated with higher odds of low RHGS. Both overweight and obese status, compared to normal weight, were related to significantly higher odds of low RHGS in both boys and girls (Table 4). Similar associations were observed for category of abdominal obesity. Boys and girls with risk of abdominal obesity had 3.3 (1.6–6.6) and 4.1 (1.8–9.3) increased odds of low RHGS, respectively, compared to boys and girls with normal WC. Those with abdominal obesity, had 8.5 (3.4–21.4) and 6.5 (2.0–21.3) increased odds of low RHGS for boys and girls, respectively, compared to boys and girls with a normal WC.

**Table 4:** Adjusted associations of low RHGS^a^ (kg/kg/m^2^) with nutritional status (model 1) and abdominal obesity (model 2) among Chilean adolescents, stratified by sex.

Variables	Boys^b^	Girls^c^
	OR (95% CI)	OR (95% CI)
**Nutritional status (Model 1)**		
Normal weight	Reference	Reference
Underweight	0.9 (0.3–3.5)	1.2 (0.2–8.2)
Overweight	3.5 (1.8–7.1)	3.2 (1.4–7.7)
Obese	3.9 (1.4–10.7)	9.1 (2.9–28.2)
**Abdominal obesity (Model 2)**		
Normal WC	Reference	Reference
At risk	3.3 (1.6–6.6)	4.1 (1.8–9.3)
Abdominal obesity	8.5 (3.4–21.4)	6.5 (2.0–21.3)

^a^ < 25^th^ percentile, <1.09 (boys) and <0.96 (girls) (kg/kg/m^2^).

^b^ Adjusted for socioeconomic level and physical activity.

^c^ Adjusted for socioeconomic level, physical activity and age.

## Discussion

Among our sample of Chilean adolescents (average age 13y) of low-to-medium socioeconomic level, 33% were overweight/obese, 27.5% were at risk for abdominal obesity, and 30.8% were sedentary. Overall, adolescents had an HGS of 26.9 kg and 1.25 kg/kgm^2^ RHGS. In multivariable analyses, we observed a clear inverse relationship between category of adiposity and RHGS among both boys and girls. Specifically, worse adiposity status (e.g., overweight or obese compared to normal weight) related to higher odds of low RHGS. To our knowledge, no other study has related abdominal obesity to RHGS.

In the current sample, the 50th percentile of HGS of the right hand ranged from 17.9 to 31.5 kg among boys and 17.5 to 24.1 for girls. Boys had a higher HGS compared with girls, which was most evident beginning at 14y. These values are, on average, higher than those reported in studies previously conducted among Chilean children and adolescents [33]. For example, for adolescents aged 10–11, we found average HGS of 17.9 and 17.5 for boys and girls, respectively; while Garcia-Hermoso et al. [33] (n = 2,026) reported 15.8 and 15.0 and Gomez-Campos et al. [34] (n = 4,604) reported 15.0 and 14.9, respectively. These differences may be due to socioeconomic level, as both of these samples were drawn exclusively from public schools, which tends to reflect a lower socioeconomic level, while the current sample contained adolescents from both public and private schools. Previous evidence has demonstrated a relationship between lower socioeconomic status and lower HGS [35]. Among Spanish adolescents, similar values have been reported: 14.7 to 40.4 for boys and 13.6 to 24.6 for girls [36]. In Canada (2007–2009) mean grip strength (based on the combined maximum score from both hands) among youth 6 to 10, 11 to 14, and 15 to 19y was greater in boys than girls for all age groups [37]. The variability is attributed to body composition and maturation stage [38,39]; on the other hand, sex differences in strength may also, in part, reflect differences in the activity preferences of girls and boys [40].

Many studies have found that HGS increases with age and, after a certain age, also differs by sex, with studies reporting higher HGS in boys compared to girls [35,41]. However, the age at which a sex difference appears varies. In a study conducted in The Netherlands, Ploegmarkers and colleagues found that HGS increased with age and began to differ by sex most noticeably after age 11y [38]. Our results partially corroborated these findings. We observed that HGS increased with age in both boys and girls. Boys and girls had similar HGS in ages 10–11 and 12–13 but began to differ later, which was similar to the findings of Gomez-Campos et al. who also studied Chilean children and adolescents [34]. Our results were also closely aligned with those of Bohannon et al. [41], who report that HGS in both the dominant and nondominant hands begin to statistically differ by sex starting at age 13, the age of puberty for many boys. Small differences in the age of appearance of a sex difference in muscle strength may relate to physical activity practices, or to differences in sexual maturity, height, or weight among younger children and adolescents from other ethnic groups. Specifically, for the Ploegmarkers study, the authors note that the Dutch population in general, and specifically, the provinces where the study took place, tend to be tall [38].

Some researchers have found a positive relationship between HGS, WC, sum of skinfold thickness, and BMI [42,43]. This relationship can be attributed to greater lean and fat mass [44–46]. In our study, we found the opposite, greater adiposity (measured by both nutritional status and abdominal obesity) related to lower HGS and RHGS among both boys and girls. In this case, higher adiposity may be reflective of lower functional muscle mass [47,48]. This also might explain why we did not find significant differences, at the bivariate level, in HGS for level of abdominal obesity for specific age groups stratified by sex—even though we did observe significant differences in adolescents overall. While nutritional status reflects body mass (bone, fat and lean mass), abdominal obesity is more reflective of fat tissue in a specific area. Thus, regardless of if there is more muscle mass (reflected in a higher BMI category and, to a lesser degree, higher WC), low functional muscle mass may relate to lower strength.

On the other hand, there is evidence, as with our results, that low HGS is inversely associated with age-related unhealthful weight gain, excessive abdominal fat, and obesity-related comorbidities [49–52]. In addition to finding that visceral fat was associated with low HGS in adult men and women with type II diabetes, Murai and colleagues found that participants with low muscle quality had greater odds of cardiovascular diseases [53]. Similarly, in a pediatric population, Ramírez-Vélez and colleagues used HGS adjusted for body weight for determining cardiometabolic risk [54].

Abdominal adiposity measured by WC seems to be a better predictor than BMI at identifying individuals at risk of an accelerated loss in muscle strength [55–57]. Fatty infiltration of muscle is associated with reduced strength [58], but increasing central adiposity could also influence muscle function through increased levels of inflammation and insulin resistance [59], with fat playing an inert role in muscle strength [60].

The concepts of dynapenic obesity (low muscle mass and power in the presence of obesity), and the co-occurrence of dynapenia and abdominal obesity (dynapenic abdominal obesity) are beginning to be discussed in adult populations, as they have been shown to predict functional decline and other health outcomes [61]. Identifying dynapenia in pediatric populations might be even more important, not only because it has been related to cardiometabolic risk, but because it may represent the beginning of functional limitations in motor activities, increases in the risk of injury during physical activity, and tendencies towards a sedentary lifestyle. Thus, determining HGS adjusted for body size and sex and comparing values to similar populations may be clinically valuable [62]. In addition, improving strength among pediatric populations would, in turn, facilitate improvements in other areas of pediatric fitness (e.g., velocity, agility, muscle resistance, balance and flexibility), necessary for braking the epidemic of childhood obesity [63].

Our results must be interpreted considering the following limitations. First, we conducted the study in the Santiago Metropolitan Region of Chile, thus findings might not be applicable for adolescents from other cities or regions of Chile. While we were able to quantify the association between two measures of adiposity—WC and BMI—and low RHGS, we did not measure body composition, which is a more specific indicator of adiposity [64] and may have elucidated relationships between muscle versus lean mass and HGS. Also, we did not determine sexual maturity, which may be important in the interpretation of HGS [34]. Finally, future studies may benefit from objective measures of physical activity or the use of a validated instrument. Strengths of our study include the use of a relatively simple and non-invasive methodology for muscle strength measurement with standardized protocol. In addition, considering the well-established sex differences in strength, we stratified all analyses by sex. Finally, to account for developmental and body size differences in adolescents, our primary outcome of interest was RHGS.

## Conclusions

We measured the association between two markers of adiposity—category of BMI and abdominal obesity—and RHGS. We found that higher adiposity was related to greater odds of low muscle strength measured by dynamometry in a sample of Chilean adolescents. From even young ages, adiposity can negatively impact muscle ability independent of elevated muscle mass. At the individual level, results imply that muscle ability could improve with decreases in adiposity; specific exercises to improve functional muscle mass might also be recommended and incentivized. At the population level, considering the growing demographic transition in the Chilean population from a young to an aging population, along with the increasing prevalence of obesity beginning in childhood, understanding how adiposity relates to low muscle strength and risk of dynapenic or sarcopenic obesity is important for Chile and many countries facing similar public health realities.
